# Supplementary material for: Endogenous tracking reveals paralog-specific targets for phosphatidic acid generation during phospholipase C signaling in living cells
Source: J Biol Chem. 2026 Sep 7;302(10):113520. doi: 10.1016/j.jbc.2026.113520 (PMC13661226; doi:10.1016/j.jbc.2026.113520)
Supplement: Supplementary file 1 [file mmc1.pdf]

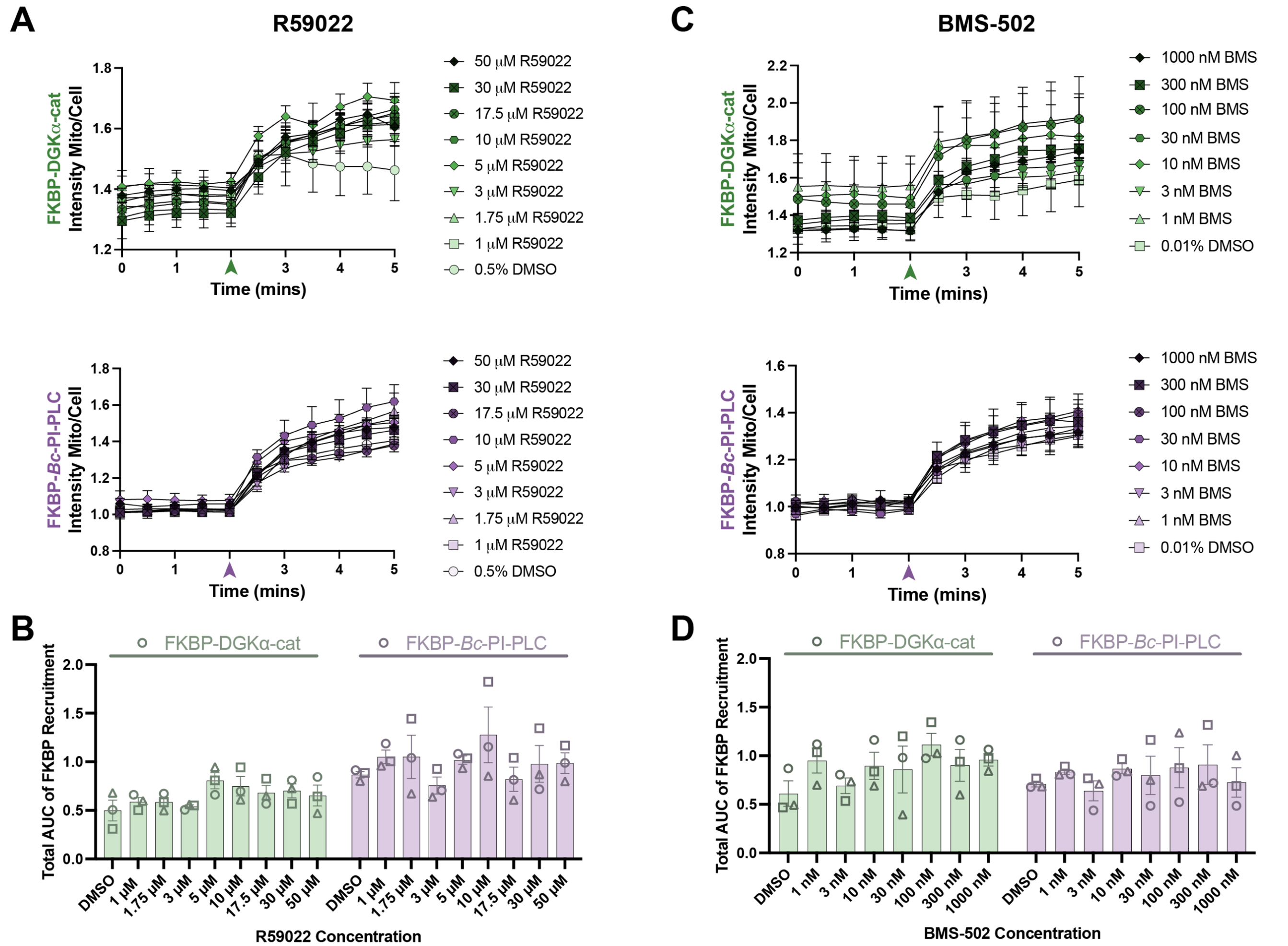

Supplemental Figure 1. DGK inhibitors do not affect recruitment of FKBP constructs to the mitochondria. (A) Recruitment of FKBP-DGKα-cat and FKBP-*Bc*-PI-PLC to the mitochondria after the addition of rapamycin and increasing doses of R59022 at the timepoint indicated by the arrow. **(B)** Total area under the curve of the recruitment shown in panel A. **(C)** As in panel A, but with increasing doses of BMS-502. **(D)** As in panel B, but showing the area under the curve data from panel C. Data in panel A-B is taken from the same cells as in Fig. 3C and data in panel C-D is from the cells in Fig. 4B. Timelapse data shown is the grand means of 3 independent experiments ± SEM. Bar graphs show the mean ± SEM of 3 independent experiments. Data from the same replicates are matched by symbol shape. 30-44 cells were analyzed.
